# Management of Acute Respiratory Failure Due to Community-Acquired Pneumonia: A Systematic Review

**DOI:** 10.3390/medsci7010010

**Published:** 2019-01-14

**Authors:** Nicolò Maria Vanoni, Manuela Carugati, Noemi Borsa, Giovanni Sotgiu, Laura Saderi, Andrea Gori, Marco Mantero, Stefano Aliberti, Francesco Blasi

**Affiliations:** 1 Fondazione IRCCS Ca’ Granda Ospedale Maggiore Policlinico, Internal Medicine Department, Respiratory Unit and Adult Cystic Fibrosis Center, 20122 Milan, Italy; nicolo.vanoni@gmail.com (N.M.V.); noemi.borsa@unimi.it (N.B.); marco.mantero@unimi.it (M.M.); francesco.blasi@unimi.it (F.B.); 2 Department of Pathophysiology and Transplantation, University of Milan, 20122 Milan, Italy; andrea.gori@unimi.it; 3 Fondazione IRCCS Ca’ Granda Ospedale Maggiore Policlinico, Internal Medicine Department, Infectious Diseases Unit, 20122 Milan, Italy; manuela.carugati@duke.edu; 4 Division of Infectious Diseases, Duke University, Durham, NC 27710, USA; 5 Clinical Epidemiology and Medical Statistics Unit, Department of Medical, Surgical and Experimental Sciences, University of Sassari, 07100 Sassari, Italy; gsotgiu@uniss.it (G.S.); lsaderi@uniss.it (L.S.)

**Keywords:** pneumonia, ventilation, CPAP, NIV, BiLevel

## Abstract

Community-acquired pneumonia (CAP) is a leading cause of mortality worldwide. CAP mortality is driven by the development of sepsis and acute respiratory failure (ARF). We performed a systematic review of the available English literature published in the period 1 January 1997 to 31 August 2017 and focused on ARF in CAP. The database searches identified 189 articles—of these, only 29 were retained for data extraction. Of these 29 articles, 12 addressed ARF in CAP without discussing its ventilatory management, while 17 evaluated the ventilatory management of ARF in CAP. In the studies assessing the ventilatory management, the specific treatments addressed were: high-flow nasal cannula (HFNC) (*n* = 1), continuous positive airway pressure (*n* = 2), non-invasive ventilation (*n* = 9), and invasive mechanical ventilation (*n* = 5). When analyzed, non-invasive ventilation (NIV) success rates ranged from 20% to 76% and they strongly predicted survival, while NIV failure led to an increased risk of adverse outcome. In conclusion, ARF in CAP patients may require both ventilatory and non-ventilatory management. Further research is needed to better evaluate the use of NIV and HFNC in those patients. Alongside the prompt administration of antimicrobials, the potential use of steroids and the implementation of severity scores should also be considered.

## 1. Introduction

The Global Burden of Disease (GBD) 2016 Lower Respiratory Infections Collaborators estimated the morbidity and mortality from lower respiratory infections: lower respiratory infections represented a leading global infectious cause of mortality in all age groups (2,377,697 deaths, 95% UI 2,145,584–2,512,809), including in children younger than 5 years (652,572 deaths, 586,475–720,612) and among adults older than 70 years (1,080,958 deaths, 943,749–1,170,638) [1]. These estimates show that despite advances in prevention and therapies, lower respiratory tract infections remain a major clinical and public health issue [1,2]. Community-acquired pneumonia (CAP) is defined as pneumonia presenting before hospitalization or within 48 h from hospital admission. Community-acquired pneumonia is a leading cause of emergency department (ED) visits and hospitalizations worldwide (e.g., 423,000 ED visits occurred in 2014 in the United States) [3]. Furthermore, CAP may be characterized by a severe clinical presentation, requiring a high level of care, such as admission to an intensive care unit (ICU) [4]. It has been estimated that CAP accounts for approximately 6% of all ICU admissions, with an ICU mortality of 35%, and overall in-hospital mortality of 50% [5].

Several scores have been developed to classify pneumonia severity and to evaluate the hospitalization need. The Pneumonia Severity Index (PSI) and the CURB65 are among the most widely used. Originally developed to predict 30-day mortality in CAP patients [6,7], they are also used to assess the need for hospital admission in accordance with the Infectious Diseases Society of America/American Thoracic Society guidelines (IDSA/ATS) for CAP, although some concerns have been recently raised [8,9]. Once the patient is admitted, identification of the most appropriate care setting (i.e., general ward vs. ICU) is key. IDSA/ATS 2007 CAP guidelines suggest major and minor criteria for ICU admission, with major criteria being the need for mechanical ventilation and the presence of septic shock requiring vasopressors [8]. These two criteria also reflect the two most relevant CAP complications: acute respiratory failure (ARF) and sepsis, which are associated with high mortality rate. Since validated severity scores are of limited use in fast-paced environments (e.g., ED), the presence of sepsis and/or respiratory failure may be used to assess CAP severity and the need for hospitalization [10].

ARF can occur in 58–87% of patients with severe CAP [11]. ARF, characterized by an impaired gas exchange between the lungs and the blood, can be managed by administering oxygen via a nasal cannula or face mask, followed by positive pressure throughout the respiratory cycle (PEEP) in case of failure. Pressure support can be administered either through endotracheal intubation (mechanical ventilation) or a non-invasive interface (non-invasive ventilation, NIV). Intubation, however, is associated with an increased risk of ventilator-associated pneumonia (VAP), ventilator-induced lung injury, increased need of sedation which further contributes to prolonged ventilation, and complications of the upper airway related with prolonged trans-laryngeal intubation NIV could have a role in avoiding intubation and its complications [12].

A recent study on a large cohort of ICU patients in France described increased NIV use as first line therapy for ARF in CAP from 1997 to 2011 [13]. The CAP guidelines do not provide clear recommendations on NIV in the management of ARF in CAP, suggesting the need for trials, particularly in patients with chronic obstructive pulmonary disease (COPD) and acute respiratory distress syndrome (ARDS) [8,14,15,16].

While NIV showed substantial benefits in cases of COPD exacerbation, acute cardiogenic pulmonary oedema, and lung infiltrates in immunosuppressed patients [17,18,19], its prescription in CAP remains uncertain following high treatment failure. The aim of the present review is to investigate ARF management in patients suffering from CAP, with a focus on the role of NIV.

## 2. Materials and Methods

### 2.1. Data Sources and Searches

Peer-reviewed articles on ARF in CAP were selected. The literature searches were run using Pubmed. The literature search was limited to articles published between January 1997 and August 2017. The search was last executed on January 2018. The following search terms were used: (acute[All Fields] AND (“respiratory insufficiency”[MeSH Terms] OR (“respiratory”[All Fields] AND “insufficiency”[All Fields]) OR “respiratory insufficiency”[All Fields] OR (“respiratory”[All Fields] AND “failure”[All Fields]) OR “respiratory failure”[All Fields])) AND ((“residence characteristics”[MeSH Terms] OR (“residence”[All Fields] AND “characteristics”[All Fields]) OR “residence characteristics”[All Fields] OR “community”[All Fields]) AND Acquired[All Fields] AND (“pneumonia”[MeSH Terms] OR “pneumonia”[All Fields])) AND ((1997/01/01”[PDAT]:“2017/08/31”[PDAT]) AND “humans”[MeSH Terms] AND English[lang]). Additional studies were manually searched and included in the list of manuscripts as “other sources”. This systematic review was conducted according to PRISMA recommendations [11].

### 2.2. Study Selection

The titles and abstracts were screened by two independent reviewers (NV and NB) using the following inclusion criteria: adult populations diagnosed with CAP and ARF. The exclusion criteria: (i) pediatric population; (ii) not a pertinent topic (ARF in conditions other than CAP; CAP not associated with ARF); (iii) reviews, meta-analysis, expert opinions, case reports, case series. When two reviewers disagreed on the article classification, a third independent reviewer (SA) resolved the tiebreak.

### 2.3. Data Extraction

The data extraction was conducted independently by two reviewers (NV and NB). The following data were extracted: authors, study design (including information on sample size, control and intervention group in case of randomized clinical trials), geography, year of publication, study aims and outcomes, and the main study results. The data synthesis and analysis: since the selected papers were highly heterogeneous, a meta-analysis was not carried out.

## 3. Results

**Figure 1:**
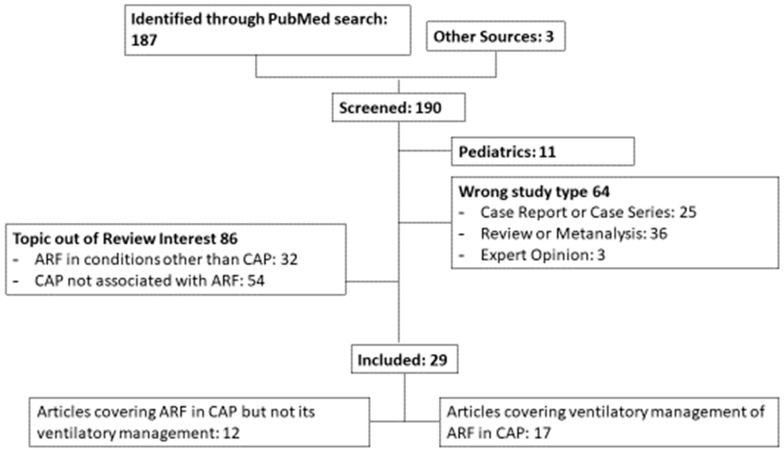
Diagrammatic overview of the review process. CAP: community-acquired pneumonia; ARF: acute respiratory failure.

A total of 187 articles were found, 29 of which were selected according to the inclusion and exclusion criteria. Three articles were included independently from the search engines (Figure 1). The papers were divided into two groups: those evaluating ARF in CAP but not its ventilatory management (*n* = 12) [10,20,21,22,23,24,25,26,27,28,29,30] and those evaluating ventilatory management of ARF in CAP (*n* = 17) [11,31,32,33,34,35,36,37,38,39,40,41,42,43,44,45,46] (Tables S1 and S2).

### 3.1. Non Ventilatory Management of Community-Acquired Pneumonia 

Two papers evaluated the administration of steroids in severe pneumonia: Chon showed that steroids were not associated with a reduction in 28-day and 3-month mortality, whereas Torres demonstrated that they could decrease the treatment failure rates [20,21].

Several authors assessed the clinical outcomes of CAP in cases of ARDS due to H1N1. In particular, Topfer and collaborators found that it was associated with the prolonged impairment of respiratory function and with a more frequent prescription of extracorporeal lung support [22]. Miyashita et al. showed that patients with ARF and *Mycoplasma pneumoniae* infection were exposed to adequate antibiotic therapy only after a significant delay [23]. In a study published by Sanz-Herrero, pneumococcal pneumonia caused by PCV-13 serotypes was associated with worse hypoxemia and a higher rate of respiratory complications [24].

Chalmers and collaborators highlighted the high negative predictive value of C Reactive Protein (CRP) on 30-day mortality; Tseng and collaborators showed an association between low procalcitonin concentrations at admission and 14-day survival. When the association between arterial PaCO_2_ and in-hospital mortality was investigated, only hypocapnia increased the risk of adverse outcomes [25]. Two other studies [10,26] evaluated the clinical factors associated with outcome in CAP patients. Aliberti et al. found that severe sepsis and ARF on admission were associated with greater mortality [10], while Kolditz et al. demonstrated that the presence of focal chest signs, home oxygen therapy, multilobar infiltrates, altered mental status, and altered vital signs at admission were independently associated with adverse outcome [26].

One study demonstrated that carriage of the C allele of pulmonary surfactant protein SP-B was associated with reduced protein levels, and, then, with a higher frequency of mechanical ventilation and of ARDS development [27]. Another study highlighted that polymorphisms of the mannose-binding leptin and its associated serine-protease (innate immunity) can predict the occurrence of complications in CAP: mannose-binding lectin insufficiency was associated with sepsis, ARF, multiorgan dysfunction syndrome, ICU admission, and death [28].

### 3.2. Ventilatory Management of Acute Respiratory Failure in Community-Acquired Pneumonia

#### 3.2.1. Non-Invasive Ventilation (NIV)

Nine papers assessed the role of NIV [11,31,32,33,34,35,36,37,38]: eight adopted an observational design (six prospective and two retrospective). They showed heterogeneous results, with a success rate ranging from 20% to 76%. NIV success predicted survival, whereas its failure was associated with increased mortality, longer ICU and hospital stay, and a higher rate of complications (e.g., sepsis). The majority of the studies chose NIV failure as the primary endpoint (i.e., need for endotracheal intubation and mechanical ventilation). Only Murad and collaborators performed a study on 209 patients using in-hospital mortality as the primary outcome and reported the highest NIV failure rate [35]. The study showing the lowest NIV failure rate (20%) was published by Nicolini in 2014 and proved a different NIV response in patients with ARF not associated with pre-existing lung or cardiac disease [36]. The same findings were described by Carrillo et al., where “de novo” ARF was associated with greater NIV failure [32]. Confalonieri et al. compared non-invasive positive pressure ventilation in CAP with standard care (oxygen therapy) [11]: NIV prevented intubation in patients with CAP associated with COPD or hypercapnic ARF.

The factors associated with an increased risk of NIV failure were: higher severity score on admission [32,35,37], deteriorating oxygenation (as indicated by a-ADO_2_, P/F or oxygenation index) or physiological parameters (respiratory and heart rate, blood pH) 1 or 2 h after NIV exposure [32,33,35,37], and radiological worsening of lung infiltrates [32,36]. Two studies compared the efficacy of NIV in ARF due to CAP or due to causes other than CAP, such as COPD and cardiogenic pulmonary oedema: the highest intubation rate was observed in patients with pneumonia [31,34].

#### 3.2.2. Continuous Positive Airway Pressure (C-PAP)

Two randomized clinical trials compared C-PAP with standard oxygen therapy. They showed superiority of C-PAP in preventing intubation following the improvement of oxygenation parameters, including respiratory rate [39,40].

#### 3.2.3. High-Flow Nasal Cannula (HFNC)

Roca and collaborators derived an indicator to assess the need for mechanical ventilation in CAP patients treated with HFNC [43]. The indicator, named ROX index, was found to be superior to the respiratory rate or P/F ratio to predict HFNC success at 12 h from ICU admission. Specifically, a ROX index greater or equal to 4.88 at 12 h from ICU admission was associated with a lower likelihood of intubation.

#### 3.2.4. Invasive Mechanical Ventilation (IMV)

When NIV and C-PAP fail, IMV is required. In patients undergoing IMV, risk factors associated with adverse outcomes were: high severity score at admission, shock, acute renal failure, severe lung injury (hypoxemia index), and limited improvement of the P/F ratio between admission and at 48 h of IMV [41,42,44,45,46]. A worse outcome was described in cases of pre-existing comorbidities [41]. One study compared mortality in ARF due to CAP or other causes, but no differences were found [42]. 

## 4. Discussion

Our systematic review showed an unexpected disproportion between the epidemiological and clinical relevance of ARF in CAP and the paucity of studies evaluating its management. Despite this, our review raised several important points regarding the management of ARF in CAP.

Recent decades have seen the progressive use of C-PAP and NIV as first line ventilatory therapies for the treatment of respiratory failure in the setting of CAP [47]. While the beneficial role of C-PAP is supported by two well designed randomized controlled trials [39,40], the effectiveness of NIV has not yet been demonstrated [8,48]. Specifically, the ability of NIV to prevent endotracheal intubation varies significantly according to the study analyzed (range 20–76%) [11,31,32,33,34,35,36,37,38]. The high variability in NIV success seems to be associated with the pathophysiologic mechanism sustaining respiratory failure: when COPD, hypercapnic respiratory failure, and cardiogenic pulmonary edema were present, patients benefited most from NIV. Furthermore, this systematic review highlighted that the failure of NIV in sustaining respiratory function led to an increased risk of adverse outcomes. Based on these data, a careful selection of candidates should be performed before starting NIV in order to provide adequate care to CAP patients [49,50].

High-Flow Nasal Cannula involves the delivery of heated and humidified oxygen via special devices at rates of up to 60 L/minute in adults. In patients with respiratory distress or failure, HFNC may be better tolerated than oxygen by face mask in terms of comfort and has been associated with a decreased respiratory rate and better oxygenation in patients of all ages and with a variety of conditions, including adults with hypoxemic respiratory failure. While HFNC is becoming increasingly widespread in clinical practice, only one study assessed its role in the treatment of ARF in CAP [43]. Specifically, Roca and collaborators attempted to identify early predictors for the need of mechanical ventilation in CAP patients presenting with acute respiratory failure and being treated with HFNC. The attempt made by Roca and colleagues is extremely relevant, since HFNC may avoid further need for mechanical ventilation in some patients, but it may also unduly delay initiation of mechanical ventilation in others and worsen their outcome [51]. The ROX index, the early predictor identified by Roca and collaborators, was characterized by a good diagnostic performance, but its external validation is still needed before its implementation in clinical practice.

Only five papers evaluated mortality-related risk factors in CAP patients requiring mechanical ventilation [41,42,44,45,46]. Unsurprisingly, comorbidities, especially in elderly patients, derangement of vital signs, and acute kidney injury were associated with adverse outcomes [52,53]. These findings mirror risk factors for in-hospital mortality in patients with sepsis [54]. The specificity and sensitivity of these variables for the identification of patients requiring mechanical ventilation at increased risk of in-hospital mortality still need to be determined. 

Another important finding is the uncertainty of the use of glucocorticoids in CAP patients. While glucocorticoids were shown not to be associated with a reduction of 28-day and 3-month mortality by a retrospective observational study [20], a randomized control trial demonstrated that the acute use of methylprednisolone compared with the placebo decreased the treatment failure rates [21]. Although not included in this systematic review, it is also worth noting that the randomized controlled trial by Annane and collaborators showed a significant benefit of hydrocortisone plus fludrocortisone on 90-day all-cause mortality in patients with septic shock [55]. Considering the findings of the above studies, the excessive host inflammatory response leading to treatment failure and mortality in CAP [56], and the fact that steroids inhibit the expression of many cytokines involved in the inflammatory response associated with pneumonia [57], glucocorticoids may have a key role in the management of CAP patients [58,59,60,61].

The delayed administration of antimicrobials can lead to adverse outcomes in CAP patients. Miyashita and collaborators observed that CAP patients developing ARF were started on an appropriate antimicrobial regimen later than CAP patients not developing ARF [23]. Similar observations have been highlighted by Kumar and Ferrer in patients with severe sepsis and septic shock [62,63]. Based on the observations by Kumar and Ferrer, the Survival Sepsis Campaign recommends the early administration of intravenous antimicrobials within one hour for both sepsis and septic shock, irrespective of the source of infection. As a consequence, this recommendation plays a key role in the management of CAP patients [64].

Clinical signs and biochemical markers could be early predictors of adverse outcomes in CAP patients. Aliberti showed that ARF and severe sepsis are associated with increased mortality [10]. Kolditz found that focal chest signs, home oxygen therapy, multi-lobar infiltrates, altered mental status, and altered vital signs are predictors of adverse outcomes [26]. CRP had a high negative predictive value for 30-day mortality in the study by Chalmers [29], whereas the procalcitonin levels (PCT levels) at enrollment correlated well with 14-day mortality in the study by Tseng and collaborators [30]. So far, several prognostic indices have been developed to risk-stratify acutely ill patients and CAP patients [6,65,66]: should CRP and PCT be included in new scores or in already available scores for CAP patients? Yang and collaborators proposed a new severity index for ICU patients which included CRP and PCT [67]. Although characterized by a good diagnostic performance, this score does not provide an immediate result due to unavoidable laboratory turnaround times; it was studied on an ICU and not on a CAP population, and, to the best of our knowledge, has not yet been externally validated. Further research is needed to fill this knowledge gap.

Finally, it is well known that an older age, chronic comorbidities, impairment of the immune system, and lifestyle factors may be associated with an increased severity of CAP presentation. This review also highlighted that genetic determinants, such as the allelic variants of pulmonary surfactant protein SP-B and deficiency in mannose-binding leptin 2, can contribute to explaining the clinical variability of CAP presentation and outcome; however, the investigation of these genetic determinants in clinical practice needs further discussion. 

Several limitations of the present study should be raised. Non-English articles were excluded. Articles were included if they presented data on ARF in CAP: sepsis studies including the management of CAP could have been excluded.

In conclusion, ARF in CAP patients may require both ventilatory and non-ventilatory management. Further research is needed to better evaluate the use of NIV and HFNC in those patients. Alongside the prompt administration of antimicrobials, the potential use of steroids and the implementation of severity scores should also be considered.

## Supplementary Materials

The following are available online at http://www.mdpi.com/2076-3271/7/1/10/s1: Table S1, articles evaluating ventilatory management of acute respiratory failure in CAP (*n* = 17); Table S2, articles evaluating acute respiratory failure in CAP but not its ventilatory management (*n* = 12).
