# Supplementary material for: Management of Acute Respiratory Failure Due to Community-Acquired Pneumonia: A Systematic Review
Source: Med Sci (Basel). 2019 Jan 14;7(1):10. doi: 10.3390/medsci7010010 (PMC6359640; doi:10.3390/medsci7010010)
Supplement: Supplementary file 1 [file medsci-07-00010-s001.pdf]

Table S1. Articles evaluating ventilatory management of acute respiratory failure in CAP (*n*=17)

First Author	Study Design	Study Setting	Country	Year of publication	Sample Size	Study population	Aim of the study	Control group for RCT	Interventional group for RCT	Outcomes	Main Results
Antonelli (31)	Prospective Observational	Multicenter	Italy	2001	354	Patients with acute hypoxemic failure due to various etiologies.	To investigate the variables of NPPV failure in patients with hypoxemic ARF.	-	-	Primary: need for endotracheal intubation and MV and risk factors associated with NIV failure. Secondary: complications and duration of NIV, length of hospital stay and ICU mortality.	Intubation was avoided in 70% of patients treated with NIV. The highest intubation rate was observed in pneumonia (50%) and ARDS (54%). These factors were independently associated with NIV failure. Patients who required intubation had higher rates of septic complications (64% vs 3%; P <0.001) and mortality.
Carrillo (32)	Prospective Observational	Single center	Spain	2012	184	Patients treated with NIV for ARF due to CAP.	To assess the characteristics and predictors of outcome of patients with CAP and severe ARF treated with NIV.	-	-	Successful NIV management: avoidance of endotracheal intubation, discharge from the ICU, patient alive and conscious for at least 24h after being transferred to the ward. NIV failure: worsening of gas exchange or respiratory distress leading to intubation or death.	NIV was successful in 63%. ARF “de novo” was associated with more frequent NIV failure than ARF superimposed on preexisting respiratory disease. Successful NIV strongly predicted survival. Predictors of NIV failure: severity score at admission (higher SOFA), worsening of radiological infiltrate and lower oxygenation after 1h, higher heart rate, lower P/F ratio and bicarbonate. Association between delayed intubation and increased mortality was found in patients with CAP and de novo ARF: the duration of NIV before intubation that best predicted mortality was ≥53h.
Carron (33)	Prospective Observational	Single center	Italy	2010	64	Patients treated with NIV for severe CAP.	To evaluate cardiorespiratory predictors of NIV failure in severe CAP.	-	-	NIV failure: need for endotracheal intubation and invasive MV.	NIV was successful in avoiding endotracheal intubation in 28 patients (44%) and failed in 36 patients (56%). Predictive factors of failure were changes in arterial oxygenation (P/F) and oxygenation index between admission and after 1h of NIV. NIV failure was also related with more severe patients, worse oxygenation and lower pH.
Confalonieri (11)	Randomized Trial	Multicenter	Italy	1999	102	Patients with severe CAP and ARF.	Compare the efficacy of NPPV delivered through a face mask with the efficacy of standard medical treatment with supplemental oxygen administration in patients with severe CAP and ARF.	Patients receiving standard treatment with oxygen supplementation delivered by Venturi mask.	Patients receiving NPPV through a face mask.	Primary outcome: developing preselected objective criteria for intubation and the need for intubation and MV at any time during the study. Secondary outcome: length of hospital stay, complications not present on admission (such as ventilator-associated pneumonia), duration of ventilatory assistance, duration of ICU and hospital stay, in-hospital survival, and 2-month survival.	Patients randomized to noninvasive ventilation had a lower rate of endotracheal intubation (21% versus 50%; p=0.03) and a shorter duration of ICU stay. Patients randomized to NPPV had a rapid, significant, and sustained reduction in RR. NIV seemed to significantly prevent intubation in patients with COPD and hypercapnic respiratory failure. Six of 16 patients without COPD (37%) and none of 12 patients with COPD randomized to NPPV required endotracheal intubation. Among patients with COPD, the arterial pH improved more rapidly.

Cosentini (39)	Randomized, open-label, controlled trial in parallel groups	Multicenter	Italy	2010	47	Patients with moderate hypoxemic ARF (RR ≤35; P/F ratio ≥200, ≤300) due to CAP.	To evaluate the efficacy of C-PAP in improving oxygenation in comparison with standard oxygen therapy.	Standard oxygen therapy	C-PAP	Primary outcome: treatment failure defined as time to reach an improvement in terms of gas exchange (P/F ratio ≥ 315) longer than 48 hours. Secondary outcomes: proportion of subjects who could maintain gas exchange improvement, frequency of adverse events and in-hospital mortality.	C-PAP therapy was the only significant predictor for reaching the end point (1,5h in the C-PAP group vs 48h in the control group P<0.001). In this study C-PAP improved oxygenation with significant rapidity and in a greater proportion of patients than in the group treated with standard oxygen. Neither cardiovascular effects nor significant adverse events related to the method were observed.
Domenighetti (34)	Prospective Observational	Single center	Switzerland	2002	33	Non-COPD adult patients with hypoxemic ARF due to severe CAP or CPE requiring NIPSV.	To compare the efficacy of NIPSV in CPE and severe CAP patients presenting with hypoxemic respiratory failure.	-	-	Achievement of the criteria for intubation, ICU stay, mortality and modification of physiological variables after 1h of NIPSV (SAPS II, Glasgow Coma Scale, RR, SpO2, complete blood gas analysis, heart rate and mean systemic arterial pressure).	Rapid improvement in oxygenation was seen in both groups (CPE and CAP). Intubation rate was higher in CAP than in those with CPE (38% vs 6% p=0.04). Duration of NIPSV, length of ICU and hospital stay was significantly shorter in the CPE group.
Murad (35)	Retrospective Observational	Multicenter	Canada	2014	209	Patients with CAP as principal diagnosis, placed on positive pressure ventilation (invasive ventilation or NIV, but not C-PAP) for at least 1h.	Assess the application of NIV in patients with CAP in a critical care setting and identify clinical and laboratory parameters that would predict NIV failure.	-	-	Primary outcome: acute hospital mortality. Secondary outcomes: NIV failure and length of ICU and hospital stay.	High NIV failure (76%). No differences in mortality nor in length of ICU and hospital stay between the initial NIV group and the initial invasive ventilation group. NIV failure was significantly associated with an increased mortality. NIV group had a lower acute severity of illness (APACHE II scores 22 vs 26, p=0.002), but higher respiratory comorbidity rate then invasive ventilation group. Significant predictors of NIV failure were found in physiological parameters measured 2 hours after the initiation of NIV (P/F ratio and pH).
Nicolini (37)	Prospective Observational	Multicenter	Italy	2016	127	Patients with severe acute respiratory failure with a diagnosis of severe CAP, who received NIV.	Assess the usefulness of NIV in severe CAP	-	-	Primary outcomes: NIV failure (considered as the need of invasive ventilation), mortality rate and their predictors.	NIV failure was 25%. More severe scores at admission (SAPS II and CURB 65), more extensive radiologic findings (Opravil score) (P < 0.003) and a higher lactate dehydrogenase (LDH) were predictive of NIV failure. Greater respiratory impairment (P/F ratio, A-aDO2, RR) at admission and after 1h of NIV was associated with higher rate of NIV failure and mortality.
Nicolini (36)	Retrospective Observational	Single center	Italy	2014	130	Patients with severe ARF (defined as P/F ratio <250) due to CAP receiving NIV treatment.	Evaluate the efficacy of NIV and factors related to its failure and mortality in Patients with CAP and severe acute respiratory failure.	-	-	The outcomes were NIV failure (defined as P/F ratio <175 after 1h of continued NIV, and/or occurrence of complications).	NIV failed in 26 patients (20%). The response to NIV treatment seemed better in patients with de novo ARF compared to patients with previous cardiac or respiratory disease, but the only significant difference was in age (47±16 vs 73±15 years, p≤0.001). Predictors of NIV failure were higher heart rate after 1h of NIV, higher A-aDO2 after 24h, higher x-ray score (opravil score) at admission.

Brambilla (10)	Open-label Randomized Controlled Trial in parallel Groups	Multicenter	Italy	2014	81	Patients with severe ARF due to pneumonia (both CAP and HAP were included).	Compare helmet C-PAP vs oxygen therapy delivered by Venturi mask in terms of reduction of the risk of meeting criteria for intubation in patients hospitalized with pneumonia.	Patients treated with C-PAP (C-PAP group).	Patients treated with oxygen therapy only (control group).	Primary endpoint: proportion of patients showing indication for intubation. Secondary endpoint: in-hospital mortality, length of hospital stay, development of adverse events, and improvement of gas exchange.	The proportion of patients who met criteria for intubation was significantly lower in the C-PAP group than in the control group (15% vs 63%, p<0.001). Intubation rate was low in both groups, but almost 70% of patients in the control group received C-PAP after meeting the intubation criteria. Helmet C-PAP promoted a faster and greater improvement in oxygenation (in terms of P/F ratio, RR and respiratory distress). No differences were found in the length of hospital stay between the two groups. Mortality in C-PAP group was 5%, whereas in the control group mortality was 40% in patients not receiving C-PAP or positive ventilatory support, and 20% in patients treated with rescue noninvasive respiratory support.
Jolliet (38)	Prospective Observational	Single center	Switzerland	2000	24	Patients with severe CAP (without chronic lung disease) treated with NIV	Evaluate the acute effects of NIV on clinical, gas exchange and hemodynamic parameters. Assess the clinical course and outcome of these patient. Analyse the nurse workload associated with NIV.	-	-	Changes in clinical, gas exchange and hemodynamic parameters. Intubation rate, mortality rate, nursing workload.	Intubation was required in 66% of the patients with a short mean delay (1,3 days). NIPSV decrease acutely respiratory rate and improved oxygenation, but no effects were found on PaCo2. The outcomes (hospital/ICU stay and mortality) was significantly different between the intubated and non intubated groups. Nursing workload was lower during the first 24 hours of NIPSV then during the equivalent period of MV.
Roca (43)	Prospective observational	Multicenter	Spai and France	2016	157	CAP patients admitted to the ICU and treated with HFNC.	To describe an index that predicts the need for MV in patients with pneumonia and ARF treated with HFNC.	-	-	HFNC failure, defined as the need for MV.	The ROX index is a better tool to predict HFNC success at 12h from ICU admission, compared with respiratory rate or P/F ratio. A ROX index greater or equal to 4.88 at 12h from ICU admission is associated with lower likelihood of intubation.
Tejerina (42)	Retrospective observational	Multicenter	20 countries	2005	124	CAP patients requiring MV on the first day of admission to hospital.	To determine variables associated with increased mortality in patients requiring MV due to CAP. To determine morbidity and ICU mortality in patients mechanically ventilated due to CAP compared to patients undergoing MV due to ARF in conditions other than CAP.	-	-	Death in patients who are mechanically ventilated due to ARF in CAP.	Three factors were independently associated with death: SAPS II score >45, shock, and acute renal failure. Variables related to oxygenation and pulmonary parameters were not associated with outcome, however patients who survived showed a better trend in these variables. CAP requiring MV was not associated with increased ICU mortality and was not associated with prolonged duration of MV or prolonged ICU stay.

Pascual (41)	Historical prospective	Single center	USA	2000	144	CAP patients requiring MV.	To identify patients at highest risk of death among those requiring MV for CAP by using both pulmonary (through the development of a hypoxia index) and non-pulmonary factors.	-	-	Death in patients requiring MV for CAP	Factors associated with death in patients who are mechanically ventilated due to CAP were: extent of lung injury (as expressed by the hypoxemia index), immunosuppression, the number of failing organ systems, age >80 years, preexisting prognosis <5 years. These factors were used to create a model that identifies patients with >95% of death.
Lee (45)	Retrospective observational	Single center	Korea	2007	85	CAP patients requiring MV.	To determine in-hospital mortality and its predictors for CAP patients requiring MV.	-	-	Mortality in CAP patients treated with MV	Mortality was 56% in the study population. Survivors had a statistically significant higher urine output during the first 24h of ICU stay, lower PaCO2, lower alveolar-arterial oxygen difference, higher albumin and shorter time on MV, compared to non-survivors. PaCO2 <45 mmHg, first 24h urine output >1.5 L and a high APACHE score were independent risk factors for death.
Wu (44)	Prospective observational	Single center	Taiwan	2007	62	Severe CAP patients requiring MV.	To evaluate the role of P/F ratio as a tool to predict outcome in severe CAP.	-	-	Survival in patients undergoing MV for CAP.	The difference between P/F ratio at ICU admission and after 48h of ICU stay was significantly different between survivors and non-survivors. An improvement in P/F Ratio >56 mmHg had a sensitivity of 75% and a specificity of 81% for survival. There was a significant improvement in the P/F ratio between admission and 48h of ICU stay in patients who received adequate antibiotic coverage. Mortality rate was 26.9% for patients on adequate initial antibiotics and 70% for those on inadequate initial antibiotics. The independent factors associated with hospital mortality were: P/F change between admission and 48h, shock upon admission and APACHE II score.
Nin (46)	Prospective observational	Multicenter	Chile and Uruguay	2011	96	Patients with confirmed or probable H1N1 infection causing acute respiratory failure requiring MV.	To describe H1N1 patients requiring MV.	-	-		NPPV was used in 45% patients, 77% of whom failed NIV. Bacterial pneumonia was diagnosed in 33% of cases, of which 8% during the first week (mainly due to community acquired bacteria) and 25% from the second week onward (mainly due to gram negative bacilli). ICU mortality was 50%. Most common causes of death: hypoxia, multi organ failure, and shock.

Table S2. Articles evaluating ARF in CAP but not its ventilatory management (n=12)

First Author	Study Design	Study Setting	Country	Year of publication	Sample Size	Study population	Aim of the study	Control group for RCT	Interventional group for RCT	Outcomes	Main Results
Chon (20)	Retrospective observational	Single center	Republic of Korea	2010	97	Patients with severe CAP who required MV in medical ICU.	To evaluate the clinical data in patients requiring MV for severe CAP and compare survival with and without the use systemic corticosteroids.	-	-	Primary outcome: patient mortality at 28 days and 3 months, and determination of factors related to mortality. Secondary outcome: effects of corticosteroids on the duration of MV, duration of ICU stay, and duration of hospital stay.	The use of corticosteroids in severe CAP patients requiring MV did not improve mortality at 28 days and 3 months. The duration of MV and ICU/hospital stay was not affected by the use of systemic corticosteroids treatment.
Torres (21)	Randomized, double-blind, placebo-controlled trial	Multicenter	Spain	2015	120	Patients with severe CAP and a high inflammatory response.	To assess the benefits from corticosteroids treatment in terms of treatment failure in patients with severe CAP and high inflammatory response.	Steroids group	Placebo group	Primary outcome: rate of treatment failure. Secondary outcome: length of ICU and hospital stay, and in-hospital mortality.	Acute administration of methylprednisolone was associated with less treatment failure. No statistically significant differences were observed among secondary clinical outcomes.
Topfer (22)	Retrospective observational	Single center	Germany	2014	62	Patients with ARDS due to severe CAP	To compare the clinical course of H1N1-induced ARDS in comparison with ARDS due to other infective causes of pneumonia (non-H1N1-ARDS).	-	-	Outcomes: improvement of clinical and functional parameters evaluated at ICU admission and over course of time for the first consecutive 10 days.	Prolonged impairment of respiratory function was observed in patients with H1N1-associated ARDS. Extracorporeal lung support was significantly more often initiated and significantly longer applied in patients with H1N1-ARDS as compared with non-H1N1-ARDS ($p < 0.05$). Lenght of ICU stay was longer in H1N1 patients.
Miyashita (23)	Prospective observational comparative trial	Multicenter	Japan	2006	227	Patients with CAP due to Mycoplasma pneumoniae infection. Patients were divided into two groups: those with ARF and those without ARF.	To clarify the clinical features of severe M. pneumoniae pneumonia in adult patients admitted to an ICU.	-	-	Comparison of clinical features between ARF and non-ARF group.	A delayed administration of adequate antibiotic therapy was found in the ARF cases (9.3 vs 5.1 days $p < 0.0001$). Bilateral pulmonary infiltrates, pleural effusion, leukocytosis, elevated levels of LDH, ALT and AST, reduced level of TP and extrapulmonary complications seemed to be significantly associated with the ARF group. All ARF patients received corticosteroids with a prompt improvement of their condition.
Sin (25)	Retrospective observational	Single center	Canada	2005	2171	Patients with CAP with available arterial blood gas analysis.	To determine the association between $PaCO_2$ and the risk of in-hospital mortality among patients admitted with CAP.	-	-	Primary outcome: in-hospital mortality. Secondary outcomes: complications and length of stay.	In-hospital mortality was greater for patients with hypocapnia (odds ratio [OR] =1.9), hypercapnia (OR =3.1) and acidosis (OR =1.7). COPD was commonly present in those with hypercapnia, but it did not influence these findings. Interestingly, RR was poorly correlated with in-hospital mortality.
Chalmers (29)	Prospective Observational	Single center	Scotland, UK	2008	570	Patients admitted with a primary diagnosis of CAP	To establish if admission and day-4 C-reactive protein (CRP) can predict 30-day mortality, need for MV and/or inotropic support, and the development of complicated pneumonia.			Primary outcome: 30-day mortality Secondary outcome: need for MV, and/or inotropic support and development of complicated pneumonia (lung abscess, empyema, or complicated parapneumonic effusion).	CRP had high negative predictive values for excluding 30-day mortality, need for MV, and/or inotropic support and complicated pneumonia up to levels <100 mg/L. Failure of CRP to fall by 50% or more at day 4 was associated with increased risk of 30-day mortality, need for MV and/or inotropic support, and complicated pneumonia.
Tseng (30)	Prospective Observational	Single center	Taiwan	2008	22	Patients with ARDS caused by CAP	To evaluate the role of procalcitonin (PCT) in predicting the outcome of patients with ARDS caused by CAP.	-	-	Primary outcome: 14-day mortality	The survivor group had significantly lower PCT concentrations at baseline and up to 72h after enrolment.
Aliberti (10)	Prospective Observational	Multicenter	Italy, Spain, UK	2014	2145	Hospitalized patients with CAP	To evaluate prevalence, characteristics, risk factors, and impact on clinical	-	-	Primaty outcome: in-hospital mortality and length of stay in the hospital (LOS).	Three distinct groups of CAP patients have been identified, showing different clinical characteristics and outcomes: those with neither ARF nor severe sepsis (4% mortality), those with only ARF (9% mortality), and those with both ARF and severe sepsis on admission (26% mortality).The presence of

							outcomes of CAP according to the presence of severe sepsis and ARF.				multilobar infiltrate on CXR on admission can help in better stratifying CAP patients according to their risk of death.
Kolditz (26)	Prospective Observational	Multicenter	Germany	2015	3427	Patients with CAP	The aim of this study was to characterize ‘CAP as a medical emergency’, defined as a need for MV, vasopressor use or death occurring within the first 72h or 7 days after hospital admission.	-	-	Emergency CAP defined as requirement for MV (invasive or non-invasive, but excluding home ventilation), vasopressor support or death within 72h or within 7 days after hospital admission, respectively.	Emergency CAP is rare but of major prognostic impact, occurring in 4% of patients within 72h and 5% within 7 days after admission. 37% of patients receiving MV/VS within 7 days presented without immediate need of MV/VS on admission but had a significantly higher 30-day mortality rate, which was highest in emergency CAP developing between days 4 and 7. Independent predictors of emergency CAP within 72h were the presence of focal chest signs, home oxygen therapy, multilobar infiltrates, altered mental status and altered vital signs (hypotension, raised respiratory or heart rate, hypothermia).
Quasney (27)	Prospective Observational	Single center	USA	2004	402	CAP patients	To test the hypothesis that adults with CAP who subsequently require MV or develop ARDS or sepsis will have a higher frequency of the CC genotype at the pulmonary surfactant protein SP-B + 1580 site compared with adults with CAP who do not develop these complications.	-	-	The need for MV; the achievement of criteria for ARDS and/or for septic shock.	Carriage of the C allele was associated with a diminished activity of pulmonary surfactant protein SP-B. A higher frequency of patients with the CC or CT genotype at the SP-B + 1580 site required MV, developed ARDS or severe sepsis compared with patients with other genotypes.
Sanz-Herrero (24)	Retrospective Observational	Single center	Spain	2016	65	Patients with bacteraemic pneumococcal pneumonia requiring hospitalization.	To assess if PCV13 serotypes are associated with worse respiratory outcome in a vaccine-naïve population with bacteraemic pneumococcal pneumonia.	-	-	Outcomes: the development of suppurative, respiratory and systemic complications, the need of ICU admission.	Different outcome of bacteraemic pneumococcal pneumonia depending on pneumococcal serotype. Patients infected with vaccine serotypes showed worse hypoxemia with lower levels of P/F ratio and showed significantly more respiratory complications when compared with those infected with non-vaccine serotypes.
Garcia-Laornen (28)	Prospective observational case-control study	Multicenter	Spain	2008	848	Hospitalized patients with CAP	To analyze the significance of MBL2 and MASP2 variability in the severity and outcome of CAP.	-	-	CAP severity and clinical outcomes	MBL2 insufficiency seemed to be associated with the development of sepsis, acute respiratory failure, multiorgan dysfunction syndrome, ICU admission, and death.

Legend

A-a DO_2: alveolo-arterial oxygen difference; ALT: alanine aminotransferase; AST: aspartate aminotransferase; ARDS: acute respiratory distress syndrome; ARF: acute respiratory failure; CAP: community-acquired pneumonia; C-PAP: continuous positive airway pressure; CPE: cardio-pulmonary edema; CRP: C-reactive protein; HFNC: high-flow nasal cannula; ICU: intensive care unit; LDH: lactate dehydrogenase; MASP2: mannose binding protein associated serine protease 2; MBL2: mannose binding lectin 2; MV: mechanical ventilation; NIV: non-invasive ventilation; NPPV: non-invasive positive pressure ventilation; NIPSV: non-invasive pressure support ventilation; PCT: procalcitonin; PCV 13: pneumococcal conjugated vaccine; P/F ratio: ratio of arterial oxygen partial pressure to fractional inspired oxygen; RR: respiratory rate.
